# Global trends and collaborations in bortezomib-induced neurotoxicity research: a bibliometric analysis (2002–2024)

**DOI:** 10.3389/fphar.2025.1584383

**Published:** 2025-05-14

**Authors:** Pin Wang, Jiaqi Liu, Lingling Cheng, Weiying Gu, Yunlong Tang, Yu Shao

**Affiliations:** ^1^ Yancheng Third People’s Hospital, Affiliated Hospital 6 of Nantong University, Yancheng, Jiangsu, China; ^2^ Department of Clinical Medicine, Nantong University College of Medicine, Nantong, Jiangsu, China; ^3^ Department of Oncology, Yancheng Traditional Chinese Medicine Hospital Affiliated to Nanjing University of Chinese Medicine, Yancheng, Jiangsu, China; ^4^ Department of Hematology, The Third Affiliated Hospital of Soochow University, Changzhou, Jiangsu, China

**Keywords:** bortezomib, neurotoxicity, bibliometrics, hematological malignancies, research trends

## Abstract

**Objectives:**

Hematological malignancies pose significant health challenges, with bortezomib emerging as a key therapeutic agent. However, its use is complicated by neurotoxicity, a side effect that urgently requires mitigation strategies. This study aims to provide a comprehensive bibliometric analysis to map the intellectual structure, historical trends, and emerging research fronts in the field of bortezomib-induced neurotoxicity.

**Methods:**

We conducted a systematic search in the Web of Science Core Collection, capturing literature from 2002 to 2024. Articles written in English were selected for analysis. Bibliometric analysis was performed using VOSviewer, CiteSpace, and R 4.3.3 to visualize collaborations, keyword co-occurrences, and bibliographic coupling.

**Results:**

The overview of this study reveals a rich tapestry of academic engagement encompassing 745 publications. The USA and China were the most productive countries, with Harvard University and the Dana-Farber Cancer Institute leading in institutional contributions. The New England Journal of Medicine stood out as the most influential journal. Prominent authors like Richardson Paul G. showcased the highest impact, with central research themes focusing on therapeutic approaches and peripheral neuropathy.

**Conclusion:**

This bibliometric analysis provides a detailed overview of the research landscape in bortezomib-induced neurotoxicity, identifying the USA and China as leading contributors and highlighting the central focus on therapeutic strategies and peripheral neuropathy management. These findings emphasize the need for global collaboration to develop effective mitigation strategies for neurotoxicity and improve patient outcomes. Insights from this study can guide future research efforts and inform clinical practices in managing bortezomib-related side effects.

## Introduction

Hematological malignancies, such as lymphoma and multiple myeloma, are marked by the uncontrolled proliferation of abnormal blood cells, leading to clinical manifestations like anemia, increased infection susceptibility, and bleeding disorders (Medical Masterclass Contributors, 2019; Jaffe, 2019). These malignancies present a considerable global health challenge, with incidence rates escalating (Zhang et al., 2023). This underscores the urgent need for aggressive therapeutic intervention (Ding et al., 2022; Rosenquist et al., 2023). In this context, bortezomib, a first-in-class proteasome inhibitor, has emerged as a pivotal component in the treatment of multiple myeloma. Its distinctive mechanism of action facilitates the degradation of essential proteins implicated in cell survival and proliferation (Syed, 2023). Bortezomib enhances existing treatments and offers significant benefits in combination regimens, improving the therapeutic landscape for patients with this challenging disease (Mateos et al., 2020).

While bortezomib has revolutionized the treatment of hematological malignancies, its use is frequently complicated by neurotoxicity, particularly peripheral neuropathy (Velasco et al., 2019; Badros et al., 2007). This side effect, ranging from mild sensory disturbances to debilitating pain, significantly impacts patients’ quality of life and can lead to treatment discontinuation (Burgess et al., 2021). Despite ongoing research into the mechanisms and management of bortezomib-induced neurotoxicity, the growing body of literature remains fragmented, making it difficult to identify overarching trends and critical gaps in knowledge (Meregalli, 2015).

To address this challenge, bibliometric analysis offers a systematic approach to mapping the intellectual structure, identifying influential studies, and uncovering emerging trends in the field (Cooper, 2015; Ninkov et al., 2022). Bibliometric indicators, when used alongside Altmetric analysis, provide a more holistic evaluation of research impact by incorporating social and public attention metrics in addition to traditional academic citations. The use of Altmetric analysis has gained prominence in recent years as a complementary tool to assess the broader influence of scholarly work, as demonstrated in studies such as Bagcier et al.’s analysis of the most-cited articles on ankylosing spondylitis (Bagcier et al., 2021).

However, no previous bibliometric or Altmetric analysis has specifically focused on the domain of bortezomib-induced neurotoxicity. This study aims to fill this gap by providing a comprehensive overview of global research efforts, highlighting clinical and research priorities to improve patient outcomes.

## Materials and methods

### Strategies for literature searching and data collection

A systematic search was conducted in the Web of Science Core Collection (WoSCC) on 4 September 2024, encompassing the period from 2002 to 2024 to identify pertinent literature. The following exact search string was used: (TS= (Bortezomib OR Velcade OR PS 341 OR LDP 341)) AND (TS=(neurotoxic* OR “peripheral neuropathy” OR “peripheral nerve toxicity”)). This search was limited to articles written in English to ensure accessibility and relevance to the study’s objectives. Full records and cited references were extracted in plain text format to enable a comprehensive analysis of the retrieved documents. The extracted data included details on the country or region of origin, the affiliated institution, the journal of publication, publication and citation dates, author details, and keywords, which were meticulously analyzed to achieve the study’s objectives.

### Statistical analysis

Three software tools were employed due to their robust capabilities in statistical analysis and data visualization: VOSviewer (version 1.6.20), CiteSpace (version 6.3.R1), and R (version 4.3.3). VOSviewer was used to map institutional collaborations and co-authorships, facilitating the exploration of complex academic networks (van Eck and Waltman, 2010). Keyword co-occurrence analysis was conducted using VOSviewer, and keyword bursts were identified with CiteSpace. Duplicate removal during data preparation was performed using software-assisted methods to ensure data accuracy and consistency. The bibliometric analysis incorporated three key metrics for evaluating the scholarly impact of publications: h-index: A metric that reflects both the productivity and citation impact of an author’s publications, defined as the number of papers (h) with at least h citations each (Mondal et al., 2023); g-index: A measure of an author’s or journal’s publication performance, emphasizing highly cited articles, calculated as the largest number (g) such that the top g articles collectively received at least g^2^ citations (Ali, 2021); and m-index: A refinement of the h-index that accounts for the length of an author’s academic career, calculated as the h-index divided by the number of years since their first publication (Saba et al., 2023). This study is based on bibliometric data and does not involve human participants, animals, or sensitive personal information. Therefore, ethical approval was not required.

## Results

### An overview of publications in research of bortezomib and neurotoxicity

**FIGURE 1:**
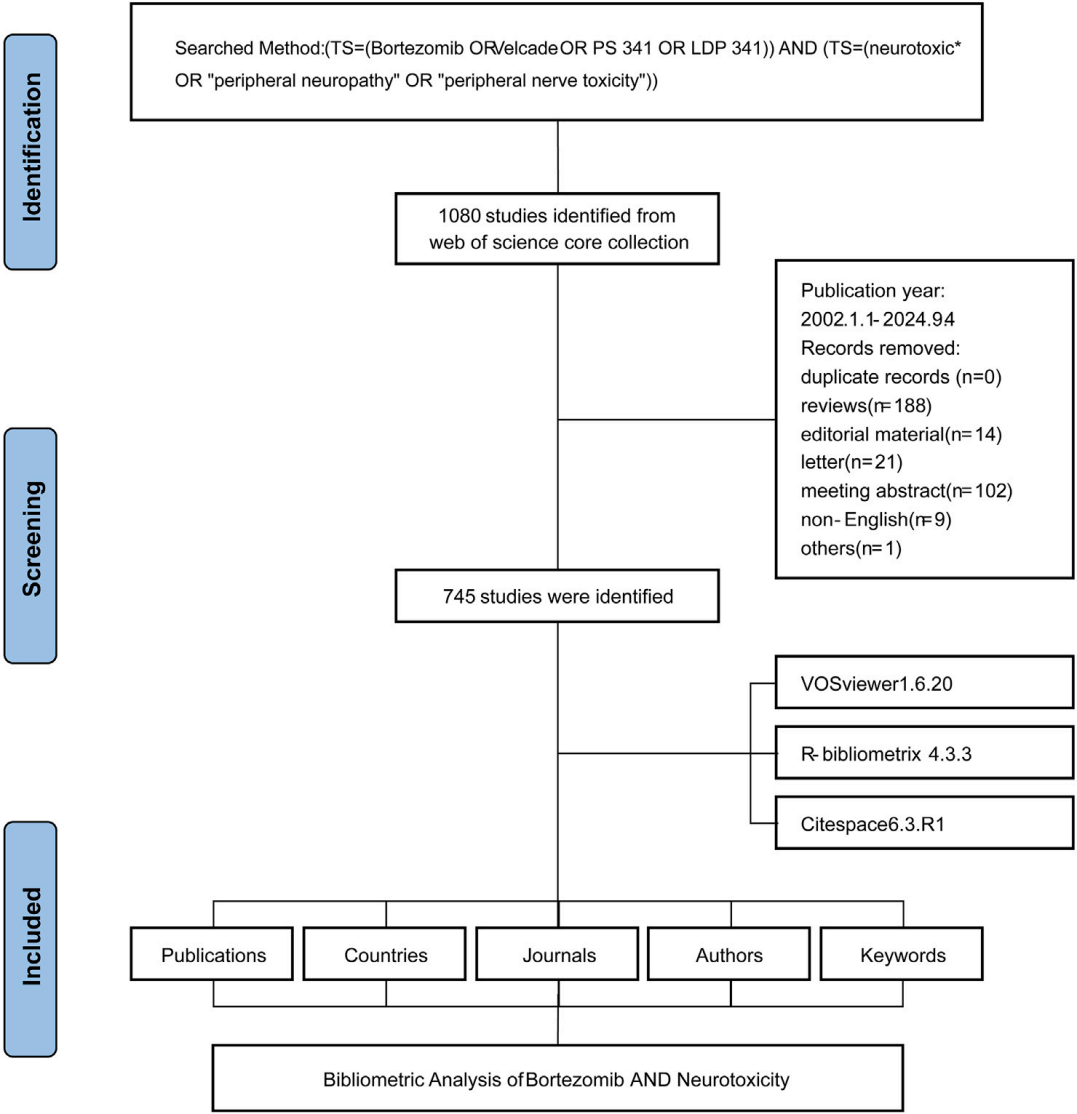
Flowchart of the literature screening process. The figure outlines the systematic process used to identify and include relevant studies, detailing the number of records retrieved, screened, excluded, and ultimately included in the bibliometric analysis.

The systematic literature screening, conducted on 29 May 2024, initially identified 1,080 studies from the Web of Science Core Collection, dating from 1 January 2002, to 4 September 2024 (Figure 1). Following the removal of duplicate records, the screening eliminated 188 reviews, 14 editorial materials, 21 letters, 102 meeting abstracts, 9 non-English articles, and 1 additional record for other reasons. This rigorous process resulted in a final selection of 745 studies.

**FIGURE 2:**
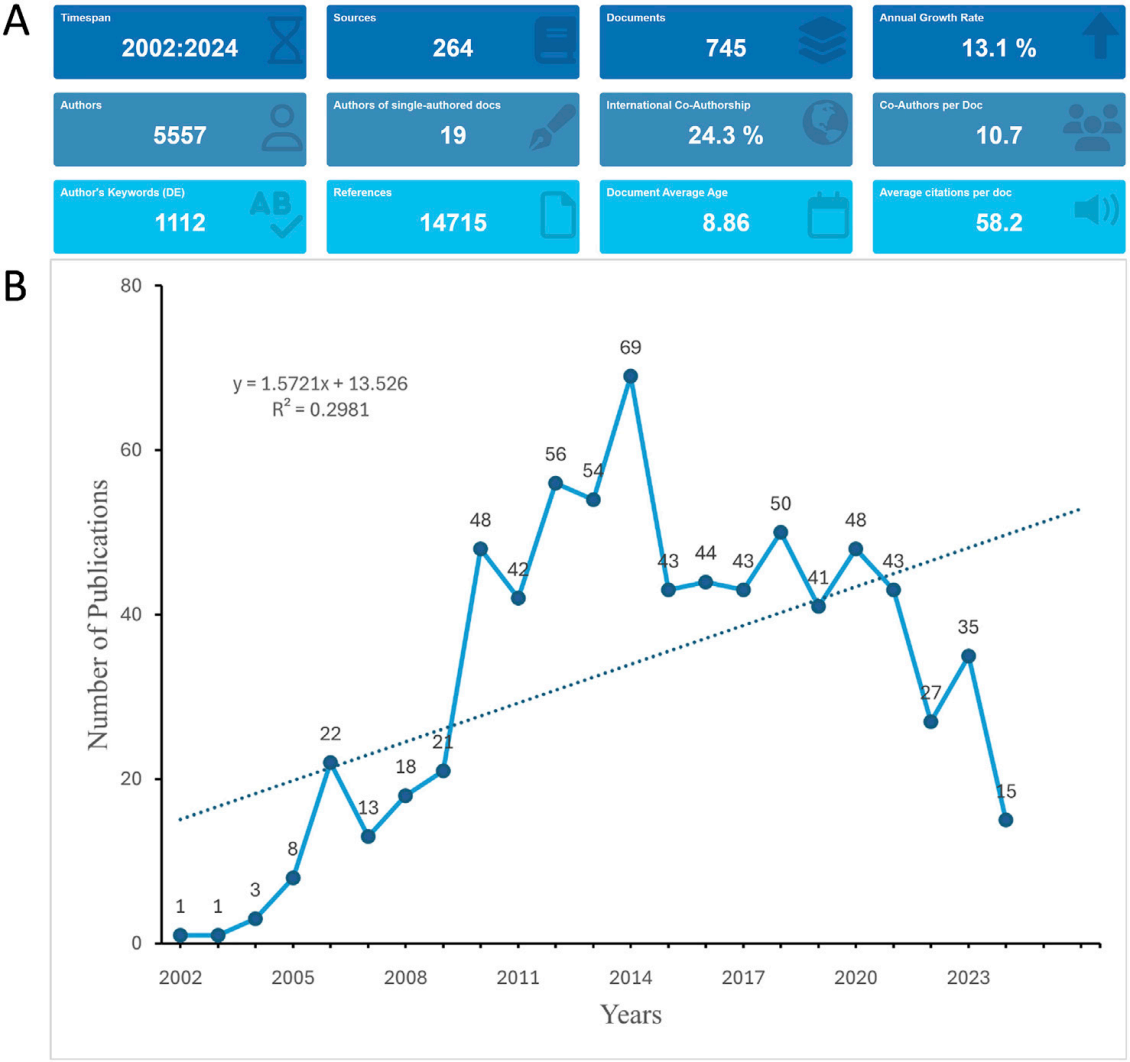
Analysis of general information. **(A)** Summary information of the included studies, providing an overview of publication types, citation metrics, and overall research trends from 2002 to 2024. **(B)** Annual number of publications on bortezomib-induced neurotoxicity. The figure highlights a peak in publication activity in 2014 and a notable decline after 2020, which could be attributed to factors such as research saturation, the COVID-19 pandemic, or shifts in funding priorities.

This study’s overview highlights extensive academic engagement, reflected in 745 publications (Figure 2A). The collective effort of 5,557 authors from 3,919 institutions across 49 countries has been harnessed to produce a global perspective on the manuscripts. These works were published in 264 journals, citing 14,715 references. The average number of authors per document is 10.7. The international co-authorship stands at 24.3%, highlighting the global nature of the research collaborations. The number of publications steadily increased after 2002, reaching a peak of 69 in 2014. For the following 6 years, approximately 40 publications were produced annually. However, since 2020, there has been a marked decrease in annual outputs (Figure 2B).

### Country and institutional analysis

**FIGURE 3:**
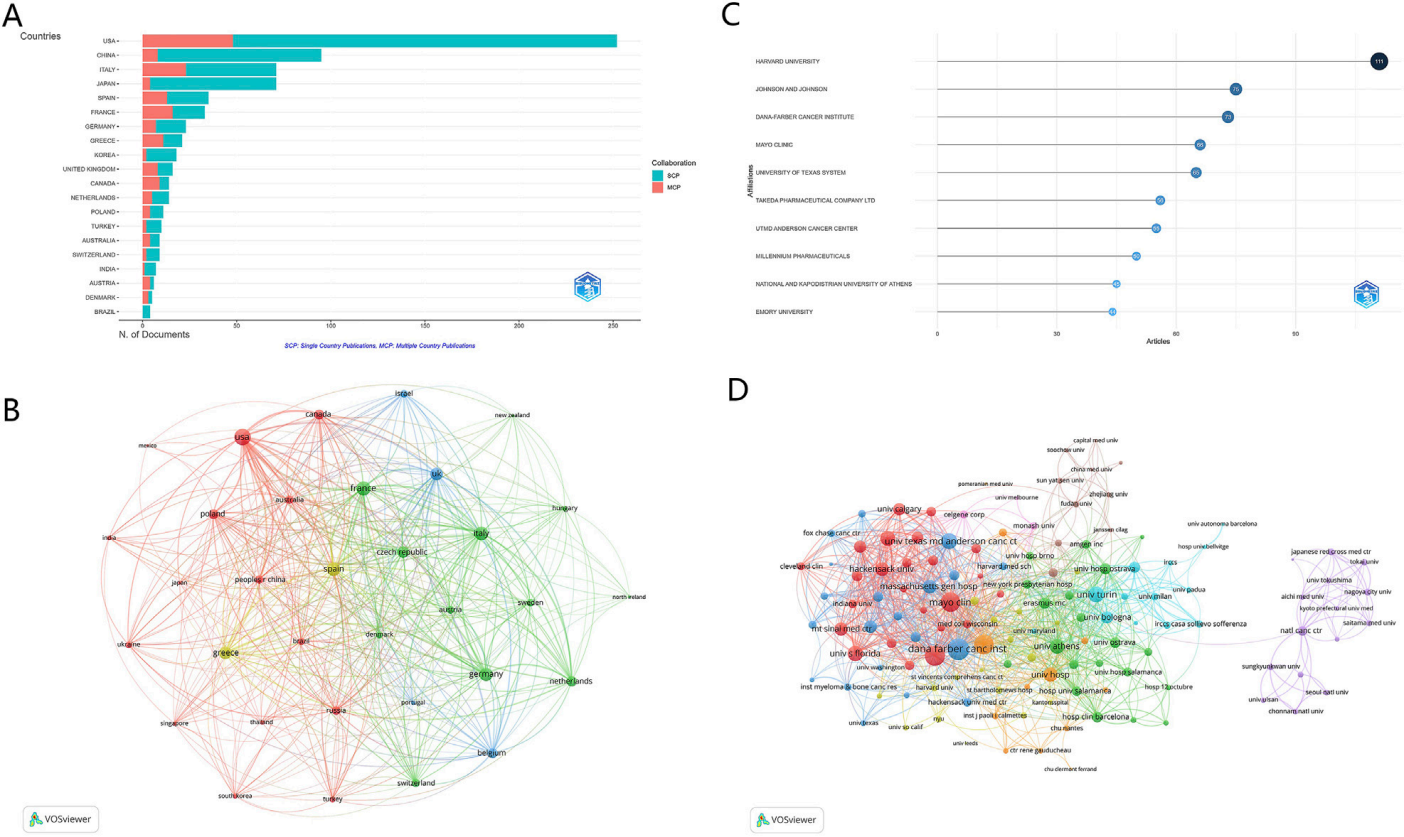
Analysis of countries and institutions. **(A)** Distribution of corresponding author’s publications by country. The United States and China lead in publication output, reflecting their dominant role in the field. **(B)** Visualization map depicting collaboration among different countries. The map highlights the USA’s central role in international research collaborations, with high total link strength indicating frequent and robust partnerships with other countries, such as France and Italy. **(C)** Top ten institutions by article count and rank. Harvard University, Johnson & Johnson, and the Dana-Farber Cancer Institute emerge as leading institutions in bortezomib-induced neurotoxicity research. **(D)** Visualization map depicting collaboration among different institutions. The map illustrates the interconnected networks of institutions, emphasizing the importance of institutional partnerships in advancing research.

The detailed profile of leading countries is displayed in Figure 3A and Supplementary Table S1, with the USA and China standing out as the most productive countries, contributing 252 and 95 articles, respectively. The USA commands the highest total publications (TP) and total citations (TC), with 1,361 and 19,928, respectively. Despite a lower publication volume, China’s research has a substantial impact, ranking fifth in terms of TPs (349) and sixth in TC (1,466). The publications from the USA, China, Italy, Japan, and Spain account for three-fourth (72.38%) of the total output. Notably, the collaboration network depicted in Figure 3B reveals a complex matrix of international partnerships. “Total link strength” in this context refers to the cumulative strength of connections between countries based on the number and intensity of co-authored publications. It quantifies how frequently countries collaborate and the depth of these collaborations (Wu et al., 2024). For instance, the USA has the highest total link strength (389), indicating its central role in international research collaborations, followed by France (301) and Italy (291).

Harvard University leads the list of top institutions in research output, with 111 articles, as highlighted in Figure 3C and Supplementary Table S2. The Johnson and Johnson comes in a strong second with 75 publications, followed closely by the Dana-Farber Cancer Institute (73 articles). Within the context of international collaborations (Figure 3D), involving a minimum of 5 articles, the Dana-Farber Cancer Institute is at the forefront with the highest number (202) of collaborations with institutions in other countries. This is trailed by Emory University with 176 collaborations and the Mayo Clinic with 172.

### Analysis of author influence and collaborative network relationships

A group of highly impactful scholars is delineated in Supplementary Table S3. Richardson Paul G. emerges as a towering figure, with an h-index of 32, a g-index of 41. His contributions to the field are further underscored by a total of 41 publications and a TC count of 8,880, which places him at the apex of author influence. Lonial Sagar, ranking a close second with a h-index of 26 and g-index of 32, has authored 32 publications that have accumulated 5,821 citations. Anderson Kenneth C. holds the third position, boasting a h-index of 24 and g-index of 25, and his 25 publications have collectively garnered 6,530 citations.

**FIGURE 4:**
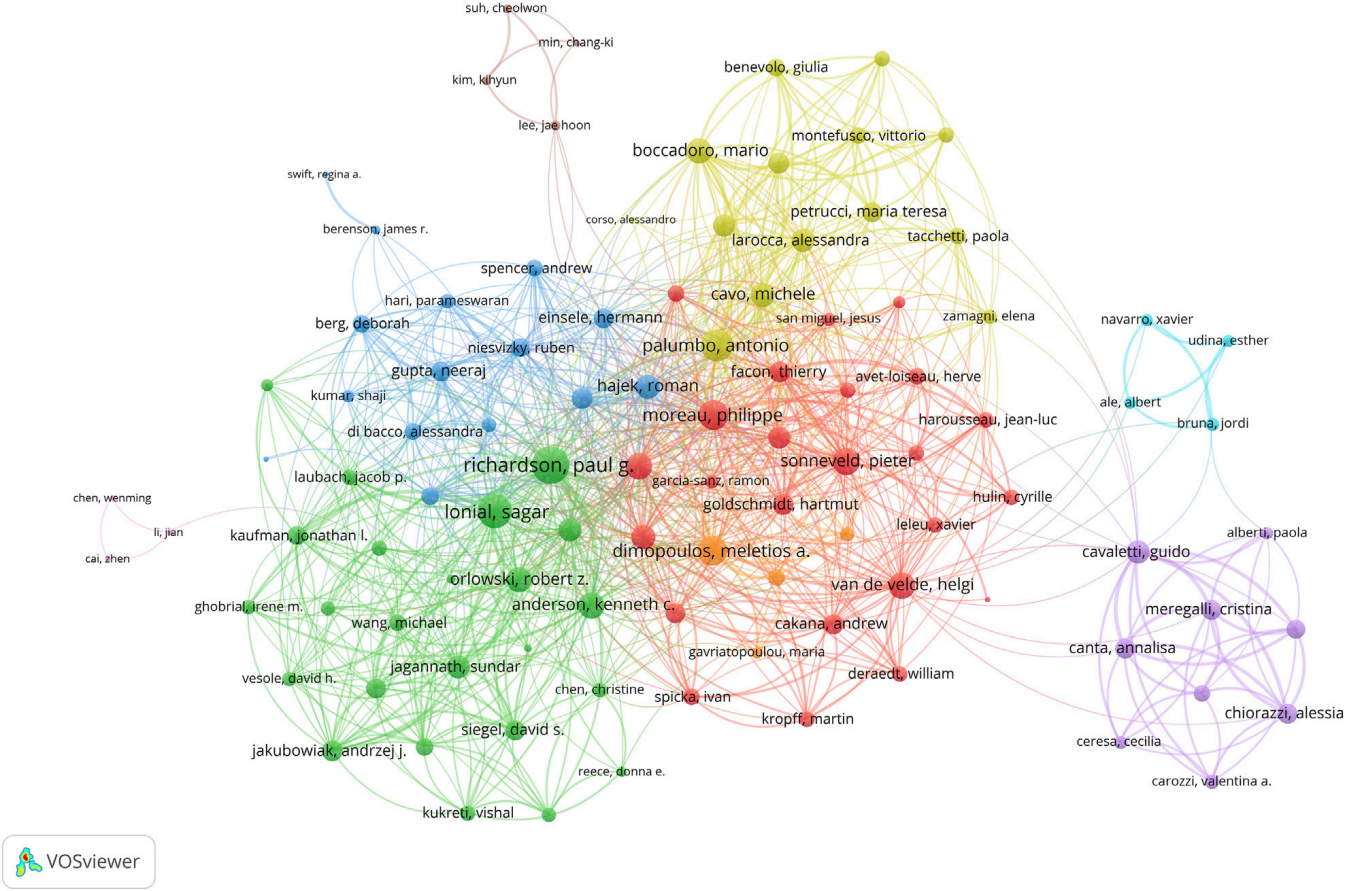
Visualization map depicting collaboration among different authors. The map showcases the author collaboration network, highlighting key contributors such as Richardson Paul G., whose extensive collaborations and high h-index underscore his significant influence in the field.

In terms of collaborative efforts (Figure 4), the data reveals an intricate web of international partnerships among authors. Richardson Paul G. leads with the highest number of collaborations with other countries, total link strength 177. This is followed by Lonial Sagar with 155 collaborations and Palumbo Antonio with 135. Among the 113 authors engaged in international collaborations with a minimum of 5 articles, these scholars stand out for their extensive collaborative efforts.

### Analysis of journal distribution

**FIGURE 5:**
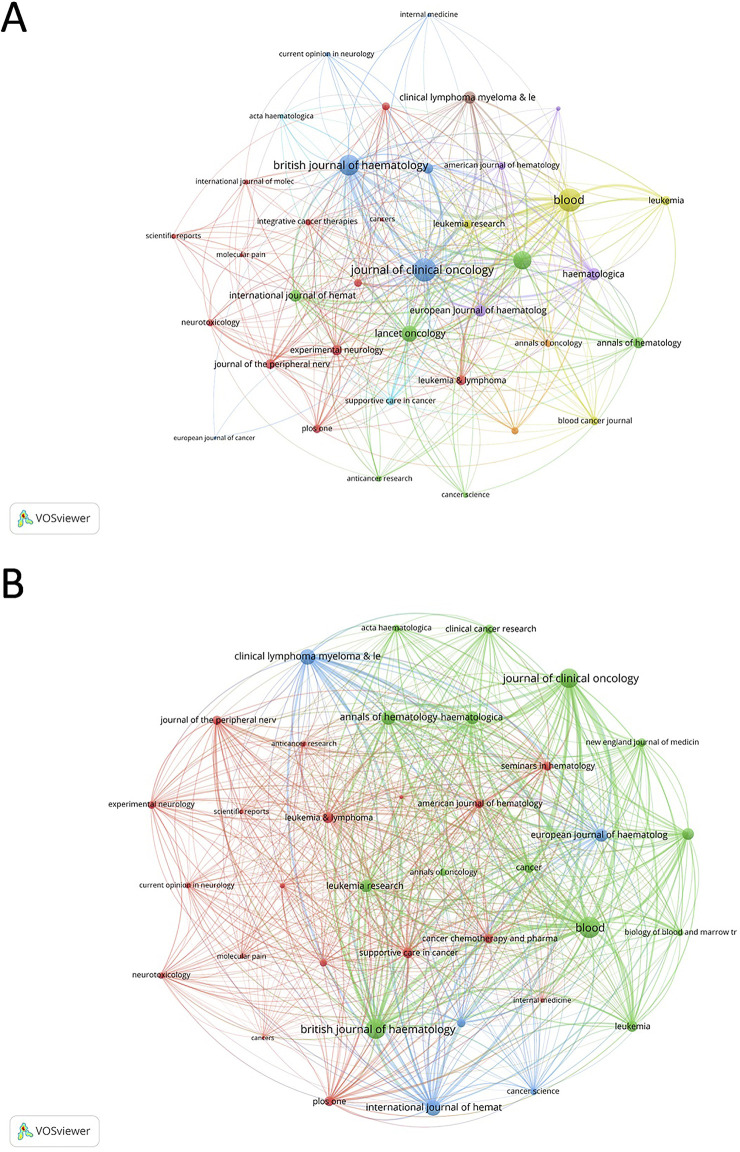
Analysis of journals. **(A)** Co-occurrence networks of journals. The network highlights journals central to the field, such as the New England Journal of Medicine, Blood, and the Journal of Clinical Oncology, which have played pivotal roles in publishing high-impact studies. **(B)** Coupling networks of journals. This network emphasizes the interconnectedness of journals that frequently reference each other, illustrating how scientific knowledge is disseminated across the field.

Blood is the most prolific journal in this domain, publishing 39 articles and garnering 3,409 citations, followed by the Journal of Clinical Oncology with 31 articles and 2,261 citations, and the British Journal of Haematology with 35 articles and 991 citations (Supplementary Table S4). Within the co-occurrence networks (Figure 5A), a total of 37 journals were identified with a minimum of 3 appearances, highlighting their frequent collaboration in published research. Prominently, Blood stood out with the highest total link strength at 487. The Journal of Clinical Oncology and the British Journal of Haematology also featured prominently, with total link strengths of 477 and 388, respectively. In the coupling networks (Figure 5B), which included 37 journals with at least 5 co-cited pairs, Blood was again a dominant player with an imposing total link strength of 18,212. This was closely followed by the British Journal of Haematology with 15,447 and the Journal of Clinical Oncology with 15,017. The New England Journal of Medicine, Journal of Clinical Oncology, and Lancet Oncology, all categorized in the Journal Citation Reports’ Q1 quartile, exhibit significant academic impact with the New England Journal of Medicine leading in Impact Factor at 96.2, followed by the Journal of Clinical Oncology at 42.1 and Lancet Oncology at 41.6.

### Analysis of top cited articles

In the domain of bortezomib research, a thorough literature analysis has been conducted. Prominently, the most cited article, “A phase 2 study of bortezomib in relapsed, refractory myeloma,” published in the New England Journal of Medicine in 2003, has garnered an extensive 2,209 citations. Following closely in terms of influence is the article titled “Bortezomib plus melphalan and prednisone for initial treatment of multiple myeloma,” which was also published in the New England Journal of Medicine in 2008, and has accumulated a total of 1,521 citations. Rounding out the top three most cited articles is the study “Lenalidomide, Bortezomib, and Dexamethasone with Transplantation for Myeloma,” published in 2017. This article has amassed 850 citations. These three articles, all published in the New England Journal of Medicine—a journal with an impressive IF of 96.2. For a more detailed examination of these articles and their bibliometric indicators, please consult the Supplementary Table S5.

### Keyword analysis

**FIGURE 6:**
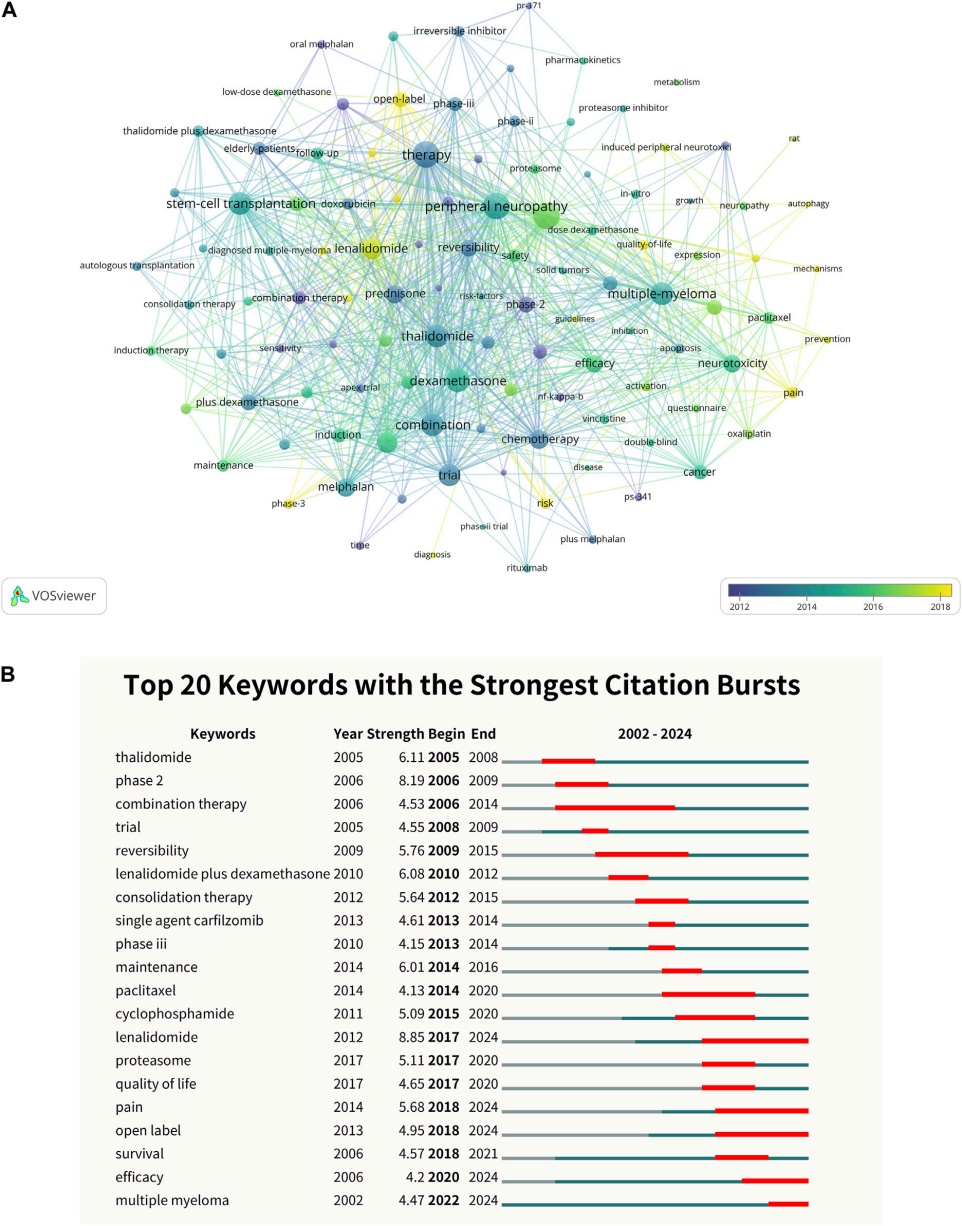
Analysis of keywords. **(A)** Visual analysis of the keyword co-occurrence network. The network identifies key research topics such as “therapy,” “peripheral neuropathy,” and “multiple myeloma,” reflecting the field’s focus on therapeutic strategies and side effect management. **(B)** Top 20 keywords with the strongest citation bursts. The figure highlights emerging trends, such as the growing interest in “lenalidomide” and “quality of life,” suggesting a shift toward patient-centered outcomes and innovative therapeutic approaches.

In the research landscape depicted in Figure 6A, the keyword “therapy” stands out as a central theme, appearing 141 times with a total link strength of 680. It is closely followed by “peripheral neuropathy,” which has 148 occurrences and a total link strength of 676, and “dexamethasone,” noted 100 times with a link strength of 519. Additionally, the terms “stem-cell transplantation” and “multiple myeloma” occur 97 and 134 times, respectively, with total link strengths of 509 and 491 (Supplementary Table S6). The analysis of burst keywords, as shown in Figure 6B, reveals intriguing trends that indicate the shifting focus within the research community. The keyword “lenalidomide” stands out with the highest burst strength of 8.85, particularly from 2017 to 2024. Following closely is the keyword “phase 2,” which has a burst strength of 8.19, especially prominent from 2006 to 2009. Additionally, “thalidomide” has a burst strength of 6.11, with significant activity from 2005 to 2008. Since 2017, there has been a noticeable clustering of keywords such as “quality of life,” “pain,” and “open label,” indicating a shift toward patient-centered outcomes in clinical trials. The term “proteasome” has also gained visibility since 2017, reflecting a growing interest in understanding the mechanisms of bortezomib and evaluating other proteasome inhibitors in clinical practice. Furthermore, the burst keyword “survival” has shown a steady increase since 2006, peaking between 2019 and 2021. Similarly, the keyword “efficacy” has gained traction since 2006, with concentration noted since 2020.

## Discussion

This bibliometric analysis aimed to explore the global trends in the field of bortezomib-induced neurotoxicity by analyzing publications from 2002 to 2024.

### General information

A notable peak in publication activity was observed in 2014, with 69 articles, indicating a significant surge in research interest. The decline in publication frequency observed after 2020 is intriguing and warrants further investigation. One plausible explanation is the saturation of research in certain areas, as foundational studies may have addressed key questions regarding bortezomib-induced neurotoxicity. Alternatively, the COVID-19 pandemic may have disrupted research activities and shifted funding priorities, diverting attention toward pandemic-related studies (Sohrabi et al., 2021). Changes in funding allocations for oncology research during this period could also have played a role. Future studies could explore these hypotheses in greater depth to better understand the factors influencing publication trends.

The analysis of national and institutional publications reveals a globally distributed research effort, with the United States and China leading in output, thereby highlighting key contributions to the scientific discourse (Attal et al., 2017; Richardson et al., 2003). The United States plays a central role in international collaborations, closely followed by France and Italy, which illustrates a robust global research network that facilitates intellectual exchange and resource sharing. Harvard University emerges as the preeminent institution for research output, consistent with its global reputation for innovation. Johnson & Johnson ranks as a significant second, while the Dana-Farber Cancer Institute stands out for its international collaborations, as evidenced by its high number of partnerships and significant citations in leading articles (Attal et al., 2017). These findings underscore the importance of global partnerships in advancing scientific research. For bortezomib-induced neurotoxicity, scholars from the United States and China substantially contribute to the scientific discourse. Notably, Richardson Paul G. possesses an h-index of 32, underscoring his significant contributions to this area, including a pivotal phase 2 study (Richardson et al., 2003; Voorhees et al., 2020; Grosicki et al., 2020; Gulla et al., 2021). His leadership in international collaborations exemplifies a broader trend in which impactful research emerges from global partnerships. This analysis elucidates how a select group of leading scholars profoundly shapes the global research landscape.

The journal landscape in the field of bortezomib-induced neurotoxicity is marked by a select group of influential publications that have played a pivotal role in shaping the scientific narrative. Notably, the New England Journal of Medicine has emerged as a preeminent outlet for this research, as evidenced by the fact that the 4 most-cited articles in this study were all published therein (Attal et al., 2017; Richardson et al., 2003; San Miguel et al., 2008; Moreau et al., 2016). This underscores the journal’s reputation for publishing high-impact studies that not only advance medical knowledge but also set the agenda for future research directions. However, it is important to note that citation counts may be influenced by journal self-citation practices and citation bias, which could overemphasize the impact of certain journals. Moreover, Blood and the Journal of Clinical Oncology have made significant contributions to the field, as indicated by their centrality in the co-occurrence and coupling networks of journals (Richardson et al., 2010; Fisher et al., 2006). These journals have been instrumental in publishing a substantial body of work that has expanded the understanding of bortezomib’s effects and facilitated important dialogues on treatment strategies and management protocols.

### Keywords reflect hotspots and trends in the research field

The keyword co-occurrence analysis in this study reveals the centrality of certain concepts within the discourse on bortezomib-induced neurotoxicity. The prominence of “therapy” and “peripheral neuropathy” in the literature underscores the dual focus on treatment strategies and the management of a significant side effect associated with bortezomib use (Scott et al., 2016; Tan et al., 2019). These findings align with previous bibliometric studies, which similarly identified therapy strategies as a dominant research focus in disease-specific bibliometric analyses (Özduran and Hancı, 2022). This reflects the broader scientific community’s emphasis on developing therapeutic options that mitigate neurotoxicity while maintaining the drug’s oncological benefits (Yamamoto and Egashira, 2021). The keyword “multiple-myeloma” standing out with high occurrences and link strength reflects the disease’s focus in the body of literature under review (Scott et al., 2016). This prominence indicates extensive research efforts dedicated to understanding the disease’s pathology and developing effective treatment regimens (Scott et al., 2016).

The frequent appearance of “dexamethasone” in the literature indicates its widespread use as a component of combination therapies, likely due to its potent anti-inflammatory and immunosuppressive properties (Voorhees et al., 2020). Its presence in the network highlights the importance of corticosteroids in the treatment paradigm, potentially in efforts to ameliorate bortezomib’s neurotoxic effects. The co-occurrence analysis of keywords provides a landscape of research priorities in bortezomib-induced neurotoxicity, highlighting efforts to refine therapeutic strategies, manage side effects, and explore innovative treatments.

The prominence of “thalidomide” with a notable burst from 2005 to 2008 highlights a resurgence of interest in this compound (Oleinikovas et al., 2024). Historically associated with severe birth defects, thalidomide has been repurposed for its potential anti-cancer properties (Vargesson and Stephens, 2021). The citation burst suggests a reevaluation of thalidomide’s clinical applications, particularly in the treatment of multiple myeloma, where its immunomodulatory and anti-angiogenic effects are being scrutinized for potential therapeutic benefits (Moehler et al., 2006). The citation burst for “phase 2” (2006–2009) highlights a period of concentrated clinical development. Phase 2 trials are pivotal in determining the efficacy and dosing of new therapeutics, and this burst reflects significant investment during this critical trial phase. These findings suggest a focused effort within the scientific community to advance the drug development process, ensuring that promising compounds proceed to later stages of testing with a robust evidence base (Voorhees et al., 2023). The significance of keyword bursts, such as “lenalidomide” and “quality of life,” reflects evolving research priorities and therapeutic strategies. The burst for “lenalidomide” (2017–2024) underscores the growing interest in this therapeutic agent as a critical component in multiple myeloma treatment, highlighting its role in improving survival outcomes (Leonard et al., 2019; Flowers et al., 2020). Similarly, the emergence of “quality of life” as a burst keyword from 2017 onward marks a notable shift towards patient-centered outcomes in clinical research. This aligns with an increasing recognition that the impact of treatments on patients’ daily lives and wellbeing is as critical as traditional survival metrics (Author Anonymous, 2021). The clustering of terms such as “pain” and “open label” further emphasizes this trend, reflecting a research landscape that is increasingly focused on addressing patient needs and optimizing treatment protocols.

The steady increase in the prominence of the keyword “survival” since 2006, peaking in 2019–2021, underscores the enduring importance of survival data in oncological research. Survival remains a cornerstone endpoint in clinical trials, reflecting the ultimate goal of extending patients’ lives (Dufva et al., 2020; Zhao et al., 2022). Similarly, the keyword “efficacy,” which has been gaining traction since 2006 with a particular concentration since 2020, highlights the continuous quest for effective therapies. The intensified focus on efficacy in recent years may reflect the rapid evolution of treatment options, including immunotherapies and targeted agents, and the need to rigorously evaluate their benefits against standard care. This focus also suggests a concerted effort to optimize treatment protocols, ensuring that the most effective therapies are identified and adopted into clinical practice.

### Strengths and limitations

The study has several notable strengths. By utilizing a comprehensive dataset from the WoSCC, it provides a reliable foundation for bibliometric analysis. The systematic investigation of keyword co-occurrence and coupling networks offers a nuanced understanding of the intellectual structure and thematic evolution of the field, highlighting research priorities, collaborative efforts, and emerging trends. The identification of burst keywords further underscores shifts in research focus, offering valuable insights for guiding future directions in bortezomib-induced neurotoxicity research. Additionally, the findings emphasize the growing focus on patient-centered outcomes, such as quality of life and pain management, which reflect a more holistic approach to cancer care. These insights have significant clinical implications, as they can inform future research funding strategies and the design of clinical trials in hematology. By identifying key research hotspots, this study provides clinicians and researchers with a comprehensive foundation for developing more effective treatment protocols and improving patient outcomes in the management of bortezomib-induced neurotoxicity.

However, several limitations warrant consideration. First, this study relied solely on the WoSCC database, which may have introduced biases or excluded relevant studies indexed in other databases such as PubMed or Scopus. The rationale for selecting WoSCC was its robust citation analysis tools and inclusion of high-impact journals, making it well-suited for bibliometric studies; however, future analyses could benefit from integrating multiple databases to enhance comprehensiveness. Second, only English-language articles and keywords were evaluated, which may have excluded important contributions published in other languages. Third, the time frame of the search (2002–2024) constrains the scope of the analysis, potentially overlooking the most recent developments in this rapidly evolving field. Fourth, duplicate removal and data cleaning were performed using automated, software-assisted methods without manual screening or quality assessment. This reliance on machine-based filtering may have introduced errors or overlooked nuances in study selection. Future studies could incorporate manual quality checks to mitigate this limitation.

## Conclusion

This study delineates key contributions, influential studies, and collaborative networks in the field of bortezomib-induced neurotoxicity. The research hotspots identified — particularly therapeutic strategies and the management of peripheral neuropathy — underline the pressing clinical need for effective treatments and mitigation of side effects. Furthermore, the findings highlight the importance of international collaborations and patient-centered outcomes, providing a roadmap for future research efforts to advance the field.

Future research should focus on addressing gaps in under-represented regions and topics. Notably, while the United States and China lead in research output, contributions from low- and middle-income countries remain limited. Expanding research efforts in these regions could provide valuable insights into the global burden of bortezomib-induced neurotoxicity and uncover novel therapeutic strategies tailored to diverse populations. Additionally, further exploration of patient-centered outcomes such as quality of life, pain management, and long-term functional recovery is crucial to align treatment strategies with the holistic needs of patients.

Building on the patterns identified in this analysis, future studies could also investigate emerging therapeutic agents like lenalidomide in greater detail, particularly their potential to mitigate neurotoxicity while maintaining efficacy in multiple myeloma treatment. Advancing clinical trial designs to incorporate endpoints related to neurotoxicity and quality of life could help prioritize treatments that balance efficacy with tolerability. Finally, leveraging advanced bibliometric tools and integrating data from multiple databases could provide a more comprehensive understanding of global research trends and further refine the roadmap for scientific progress in this field.

This study delineates key contributions, influential studies, and collaborative networks in the field of bortezomib-induced neurotoxicity. The research hotspots identified — particularly therapeutic strategies and the management of peripheral neuropathy — underline the pressing clinical need for effective treatments and mitigation of side effects. Furthermore, the findings highlight the importance of international collaborations and patient-centered outcomes, providing a roadmap for future research efforts to advance the field.

## Data Availability

The original contributions presented in the study are included in the article/Supplementary Material, further inquiries can be directed to the corresponding authors.
