# Supplementary material for: Global trends and collaborations in bortezomib-induced neurotoxicity research: a bibliometric analysis (2002–2024)
Source: Front Pharmacol. 2025 May 14;16:1584383. doi: 10.3389/fphar.2025.1584383 (PMC12116503; doi:10.3389/fphar.2025.1584383)
Supplement: Supplementary file 1 [file Table1.docx]

**Supplementary Table 1 Publication and Citation Profiles of Leading Countries**

| **COUNTRY** | **ARTICLES** | **FREQ** | **MCP_RATIO** | **TP** | **TP_RANK** | **TC** | **TC_RANK** | **AVERAGE CITATIONS** |
| --- | --- | --- | --- | --- | --- | --- | --- | --- |
| USA | 252 | 0.338 | 0.190 | 1361 | 1 | 19928 | 1 | 79.1 |
| CHINA | 95 | 0.128 | 0.084 | 349 | 5 | 1466 | 6 | 15.4 |
| ITALY | 71 | 0.095 | 0.324 | 487 | 2 | 4852 | 2 | 68.3 |
| JAPAN | 71 | 0.095 | 0.056 | 423 | 3 | 884 | 7 | 12.5 |
| SPAIN | 35 | 0.047 | 0.371 | 245 | 6 | 4835 | 3 | 138.1 |
| FRANCE | 33 | 0.044 | 0.485 | 383 | 4 | 4525 | 4 | 137.1 |
| GERMANY | 23 | 0.031 | 0.304 | 193 | 7 | 635 | 10 | 27.6 |
| GREECE | 21 | 0.028 | 0.524 | 94 | 12 | 1507 | 5 | 71.8 |
| KOREA | 18 | 0.024 | 0.111 | 125 | 9 | 289 | 16 | 16.1 |
| UNITED KINGDOM | 16 | 0.021 | 0.500 | 145 | 8 | 507 | 12 | 31.7 |
| CANADA | 14 | 0.019 | 0.643 | 84 | 13 | 853 | 8 | 60.9 |
| NETHERLANDS | 14 | 0.019 | 0.357 | 104 | 10 | 649 | 9 | 46.4 |
| POLAND | 11 | 0.015 | 0.364 | 95 | 11 | 369 | 14 | 33.5 |
| TURKEY | 10 | 0.013 | 0.200 | 39 | 18 | 184 | 17 | 18.4 |
| AUSTRALIA | 9 | 0.012 | 0.444 | 83 | 14 | 315 | 15 | 35 |
| SWITZERLAND | 9 | 0.012 | 0.222 | 51 | 16 | 425 | 13 | 47.2 |
| INDIA | 7 | 0.009 | 0.143 | 28 | 20 | 48 | 21 | 6.9 |
| AUSTRIA | 6 | 0.008 | 0.667 | 20 | 25 | 508 | 11 | 84.7 |
| DENMARK | 5 | 0.007 | 0.600 | 29 | 19 | 32 | 24 | 6.4 |
| BRAZIL | 4 | 0.005 | 0.000 | 25 | 21 | 7 | 27 | 1.8 |

**Supplementary Table 2 Number of articles by institution**

| **AFFILIATION** | **ARTICLES** |
| --- | --- |
| HARVARD UNIVERSITY | 111 |
| JOHNSON AND JOHNSON | 75 |
| DANA-FARBER CANCER INSTITUTE | 73 |
| MAYO CLINIC | 66 |
| UNIVERSITY OF TEXAS SYSTEM | 65 |
| TAKEDA PHARMACEUTICAL COMPANY LTD | 56 |
| UTMD ANDERSON CANCER CENTER | 55 |
| MILLENNIUM PHARMACEUTICALS | 50 |
| NATIONAL AND KAPODISTRIAN UNIVERSITY OF ATHENS | 45 |
| EMORY UNIVERSITY | 44 |
| NANTES UNIVERSITE | 41 |
| ERASMUS UNIVERSITY ROTTERDAM | 40 |
| ASSISTANCE PUBLIQUE HOPITAUX PARIS (APHP) | 38 |
| ERASMUS MC | 38 |
| UNICANCER | 38 |
| UNIVERSITY OF MILANO-BICOCCA | 37 |
| ATHENS MEDICAL SCHOOL | 35 |
| CHU DE NANTES | 34 |
| UNIVERSITY OF CALIFORNIA SYSTEM | 33 |
| UNIVERSITY OF TURIN | 33 |
| RUPRECHT KARLS UNIVERSITY HEIDELBERG | 32 |
| UNIVERSITY OF LONDON | 32 |
| JANSSEN PHARMACEUTICALS | 30 |
| HARVARD MEDICAL SCHOOL | 29 |
| UNIVERSITY OF BARCELONA | 29 |
| A.O.U. CITTA DELLA SALUTE E DELLA SCIENZA DI TORINO | 26 |
| SUN YAT SEN UNIVERSITY | 26 |
| UNIVERSITY OF TORONTO | 26 |
| INSTITUT NATIONAL DE LA SANTE ET DE LA RECHERCHE MEDICALE (INSERM) | 25 |
| UNIVERSITY SYSTEM OF OHIO | 25 |
| PRINCESS MARGARET CANCER CENTRE | 24 |
| SAPIENZA UNIVERSITY ROME | 24 |
| UNIVERSITY HEALTH NETWORK TORONTO | 24 |
| UNIVERSITY OF BOLOGNA | 24 |
| ZHEJIANG UNIVERSITY | 24 |
| CHU CLERMONT FERRAND | 23 |
| UNIVERSITE PARIS CITE | 23 |
| UNIVERSITY OF CALIFORNIA SAN FRANCISCO | 23 |
| WASHINGTON UNIVERSITY (WUSTL) | 23 |
| BERLIN INSTITUTE OF HEALTH | 22 |
| CHARITE UNIVERSITATSMEDIZIN BERLIN | 22 |
| CHU LILLE | 22 |
| FREE UNIVERSITY OF BERLIN | 22 |
| HELMHOLTZ ASSOCIATION | 22 |
| HUMBOLDT UNIVERSITY OF BERLIN | 22 |
| UNIVERSITE DE LILLE | 22 |
| CORNELL UNIVERSITY | 21 |
| WEILL CORNELL MEDICINE | 21 |
| CITY OF HOPE | 20 |
| FUDAN UNIVERSITY | 20 |

**Supplementary Table 3 Publication and Citation Profiles of High-Impact Authors**

| **AUTHORS** | **H_INDEX** | **G-INDEX** | **M-INDEX** | **PY_START** | **TP** | **TP_FRAC** | **TP_RANK** | **TC** | **TC_RANK** |
| --- | --- | --- | --- | --- | --- | --- | --- | --- | --- |
| RICHARDSON PAUL G. | 32 | 41 | 1.68 | 2006 | 41 | 2.66 | 1 | 8880 | 1 |
| LONIAL SAGAR | 26 | 32 | 1.37 | 2006 | 32 | 2.48 | 3 | 5821 | 3 |
| ANDERSON KENNETH C. | 24 | 25 | 1.26 | 2006 | 25 | 1.90 | 5 | 6530 | 2 |
| MOREAU PHILIPPE | 23 | 26 | 1.21 | 2006 | 26 | 1.43 | 4 | 5402 | 5 |
| DIMOPOULOS MELETIOS A. | 20 | 32 | 1.00 | 2005 | 32 | 2.82 | 2 | 4367 | 6 |
| PALUMBO ANTONIO | 20 | 23 | 1.05 | 2006 | 23 | 1.15 | 6 | 5455 | 4 |
| SONNEVELD PIETER | 18 | 20 | 0.95 | 2006 | 20 | 2.08 | 9 | 2292 | 22 |
| JAGANNATH SUNDAR | 17 | 20 | 0.90 | 2006 | 20 | 1.31 | 8 | 4280 | 7 |
| CAVALETTI GUIDO | 16 | 20 | 0.89 | 2007 | 20 | 2.06 | 7 | 1224 | 40 |
| VAN DE VELDE HELGI | 16 | 17 | 0.94 | 2008 | 17 | 1.20 | 12 | 3738 | 8 |
| BLADE JOAN | 15 | 16 | 0.79 | 2006 | 16 | 1.07 | 13 | 3043 | 10 |
| BOCCADORO MARIO | 14 | 16 | 0.74 | 2006 | 16 | 0.86 | 14 | 2299 | 21 |
| CAVO MICHELE | 14 | 17 | 0.93 | 2010 | 17 | 0.85 | 10 | 2856 | 14 |
| FACON THIERRY | 14 | 16 | 0.88 | 2009 | 16 | 0.77 | 15 | 2917 | 12 |
| HAJEK ROMAN | 14 | 17 | 1.00 | 2011 | 17 | 0.88 | 11 | 1599 | 36 |
| ORLOWSKI ROBERT Z. | 14 | 16 | 0.74 | 2006 | 16 | 1.56 | 16 | 2767 | 15 |
| ESSELTINE DIXIE-LEE | 13 | 14 | 0.68 | 2006 | 14 | 1.27 | 18 | 2905 | 13 |
| MATEOS MARIA-VICTORIA | 13 | 15 | 0.68 | 2006 | 15 | 1.78 | 17 | 3155 | 9 |
| GOLDSCHMIDT HARTMUT | 12 | 14 | 0.75 | 2009 | 14 | 1.26 | 19 | 1482 | 38 |
| ORIOL ALBERT | 12 | 13 | 0.63 | 2006 | 13 | 0.76 | 21 | 2087 | 28 |

Note(s): H_index: The h-index of the journal, which measures both the productivity and citation impact of the publications. g_index: The g-index of the journal, which gives more weight to highly-cited articles. m_index: The m-index of the journal, which is the h-index divided by the number of years since the first published paper. TP: Total Publications. TP_rank: Rank of Total Publications. TC: Total Citations. TC_rank: Rank of Total Citations. Average Citations: The average number of citations per publication. PY_start: Publication Year Start, indicating the year the journal started publication

**Supplementary Table 4 Bibliometric Indicators of High-Impact Journals**

| **JOURNAL** | **H_INDEX** | **IF** | **JCR_QUARTILE** | **PY_START** | **TP** | **TP_RANK** | **TC** | **TC_RANK** |
| --- | --- | --- | --- | --- | --- | --- | --- | --- |
| BLOOD | 38 | 21 | Q1 | 2005 | 39 | 1 | 3409 | 1 |
| JOURNAL OF CLINICAL ONCOLOGY | 29 | 42.1 | Q1 | 2005 | 31 | 3 | 2261 | 2 |
| BRITISH JOURNAL OF HAEMATOLOGY | 21 | 5.1 | Q1 | 2004 | 35 | 2 | 991 | 4 |
| HAEMATOLOGICA | 13 | 8.2 | Q1 | 2005 | 13 | 9 | 436 | 8 |
| ANNALS OF HEMATOLOGY | 12 | 3 | Q2 | 2010 | 20 | 6 | 163 | 25 |
| CLINICAL CANCER RESEARCH | 10 | 10 | Q1 | 2004 | 10 | 12 | 570 | 6 |
| EUROPEAN JOURNAL OF HAEMATOLOGY | 10 | 2.3 | Q2 | 2006 | 14 | 7 | 284 | 12 |
| LANCET ONCOLOGY | 10 | 41.6 | Q1 | 2010 | 10 | 13 | 557 | 7 |
| AMERICAN JOURNAL OF HEMATOLOGY | 9 | 10.1 | Q1 | 2011 | 13 | 8 | 247 | 20 |
| LEUKEMIA | 9 | 12.8 | Q1 | 2008 | 9 | 16 | 826 | 5 |
| CANCER | 8 | 6.1 | Q1 | 2007 | 8 | 17 | 281 | 13 |
| CLINICAL LYMPHOMA MYELOMA & LEUKEMIA | 8 | 2.7 | Q3 | 2010 | 23 | 4 | 137 | 29 |
| EXPERIMENTAL NEUROLOGY | 8 | 4.6 | Q1 | 2007 | 9 | 15 | 324 | 10 |
| INTERNATIONAL JOURNAL OF HEMATOLOGY | 8 | 1.7 | Q3 | 2006 | 23 | 5 | 94 | 41 |
| LEUKEMIA & LYMPHOMA | 8 | 2.2 | Q3 | 2011 | 13 | 10 | 261 | 15 |
| LEUKEMIA RESEARCH | 8 | 2.1 | Q3 | 2006 | 10 | 14 | 111 | 35 |
| SUPPORTIVE CARE IN CANCER | 8 | 2.8 | Q1 | 2012 | 13 | 11 | 140 | 28 |
| CANCER CHEMOTHERAPY AND PHARMACOLOGY | 7 | 2.7 | Q2 | 2008 | 7 | 23 | 100 | 38 |
| JOURNAL OF THE PERIPHERAL NERVOUS SYSTEM | 7 | 3.9 | Q2 | 2008 | 8 | 18 | 249 | 18 |
| PLOS ONE | 7 | 2.9 | Q1 | 2013 | 8 | 19 | 186 | 23 |

Note(s): H_index: The h-index of the journal, which measures both the productivity and citation impact of the publications. IF: Impact Factor, indicating the average number of citations to recent articles published in the journal. JCR_Quartile: The quartile ranking of the journal in the Journal Citation Reports, indicating the journal's ranking relative to others in the same field (Q1: top 25%, Q2: 25%-50%, Q3: 50%-75%, Q4: bottom 25%). TP: Total Publications. TP_rank: Rank of Total Publications. TC: Total Citations. TC_rank: Rank of Total Citations. Average Citations: The average number of citations per publication. PY_start: Publication Year Start, indicating the year the journal started publication.

**Supplementary Table 5 List of Highly Cited Literature**

| **PAPER** | **DOI** | **TOTAL CITATIONS** | **TC PER YEAR** | **NORMALIZED TC** |
| --- | --- | --- | --- | --- |
| RICHARDSON PG, 2003, NEW ENGL J MED | 10.1056/NEJMoa030288 | 2209 | 100.41 | 1.00 |
| SAN MIGUEL JF, 2008, NEW ENGL J MED | 10.1056/NEJMoa0801479 | 1521 | 89.47 | 11.27 |
| ATTAL M, 2017, NEW ENGL J MED | 10.1056/NEJMoa1611750 | 850 | 106.25 | 17.70 |
| MOREAU P, 2016, NEW ENGL J MED | 10.1056/NEJMoa1516282 | 797 | 88.56 | 19.91 |
| MOREAU P, 2011, LANCET ONCOL | 10.1016/S1470-2045(11)70081-X | 724 | 51.71 | 9.73 |
| RICHARDSON PG, 2010, BLOOD | 10.1182/blood-2010-02-268862 | 687 | 45.80 | 6.39 |
| CAVO M, 2010, LANCET | 10.1016/S0140-6736(10)61424-9 | 678 | 45.20 | 6.31 |
| SAN-MIGUEL JF, 2014, LANCET ONCOL | 10.1016/S1470-2045(14)70440-1 | 620 | 56.36 | 10.07 |
| FISHER RI, 2006, J CLIN ONCOL | 10.1200/JCO.2006.07.9665 | 602 | 31.68 | 3.47 |
| JAGANNATH S, 2004, BRIT J HAEMATOL | 10.1111/j.1365-2141.2004.05188.x | 598 | 28.48 | 2 |
| BARLOGIE B, 2006, NEW ENGL J MED | 10.1056/NEJMoa053583 | 583 | 30.68 | 3.36 |
| SIEGEL DS, 2012, BLOOD | 10.1182/blood-2012-05-425934 | 504 | 38.77 | 6.33 |
| RICHARDSON PG, 2006, J CLIN ONCOL | 10.1200/JCO.2005.04.7779 | 488 | 25.68 | 2.82 |
| GOY A, 2005, J CLIN ONCOL | 10.1200/JCO.2005.03.108 | 440 | 22 | 3.3 |
| HAROUSSEAU JL, 2010, J CLIN ONCOL | 10.1200/JCO.2009.27.9158 | 420 | 28 | 3.91 |
| RICHARDSON PG, 2006, BLOOD | 10.1182/blood-2006-04-015909 | 419 | 22.05 | 2.42 |
| ROSIÑOL L, 2012, BLOOD | 10.1182/blood-2012-02-408922 | 388 | 29.85 | 4.87 |
| MATEOS MV, 2010, LANCET ONCOL | 10.1016/S1470-2045(10)70187-X | 368 | 24.53 | 3.42 |
| ZHAO WH, 2018, J HEMATOL ONCOL | 10.1186/s13045-018-0681-6 | 366 | 52.29 | 10.56 |
| GRISOLD W, 2012, NEURO-ONCOLOGY | 10.1093/neuonc/nos203 | 347 | 26.69 | 4.35 |
| JAKUBOWIAK AJ, 2012, BLOOD | 10.1182/blood-2012-04-422683 | 341 | 26.23 | 4.28 |
| PALUMBO A, 2010, J CLIN ONCOL | 10.1200/JCO.2010.29.8216 | 338 | 22.53 | 3.14 |
| MATEOS MV, 2010, J CLIN ONCOL | 10.1200/JCO.2009.26.0638 | 337 | 22.47 | 3.14 |
| KUMAR S, 2012, BLOOD | 10.1182/blood-2011-11-395749 | 334 | 25.69 | 4.19 |
| ARASTU-KAPUR S, 2011, CLIN CANCER RES | 10.1158/1078-0432.CCR-10-1950 | 326 | 23.29 | 4.38 |
| BRINGHEN S, 2010, BLOOD | 10.1182/blood-2010-07-294983 | 310 | 20.67 | 2.88 |
| MATEOS MV, 2006, BLOOD | 10.1182/blood-2006-04-019778 | 309 | 16.26 | 1.78 |
| RICHARDSON PG, 2014, BLOOD-a-b | 10.1182/blood-2013-11-538835 | 300 | 27.27 | 4.87 |
| ROBAK T, 2015, NEW ENGL J MED | 10.1056/NEJMoa1412096 | 299 | 29.9 | 6.54 |
| BROSS PF, 2004, CLIN CANCER RES | 10.1158/1078-0432.CCR-03-0781 | 298 | 14.19 | 1 |
| DIMOPOULOS MA, 2017, LANCET ONCOL | 10.1016/S1470-2045(17)30578-8 | 297 | 37.13 | 6.18 |
| KANE RC, 2006, CLIN CANCER RES | 10.1158/1078-0432.CCR-06-0170 | 292 | 15.37 | 1.68 |
| PALUMBO A, 2014, J CLIN ONCOL-a | 10.1200/JCO.2013.48.7934 | 279 | 25.36 | 4.53 |
| GOY A, 2009, ANN ONCOL | 10.1093/annonc/mdn656 | 271 | 16.94 | 3.3 |
| BARLOGIE B, 2007, BRIT J HAEMATOL | 10.1111/j.1365-2141.2007.06639.x | 260 | 14.44 | 2.25 |
| ORLOWSKI RZ, 2005, BLOOD | 10.1182/blood-2004-07-2911 | 259 | 12.95 | 1.94 |
| SIEGEL D, 2013, HAEMATOLOGICA | 10.3324/haematol.2013.089334 | 258 | 21.5 | 6.04 |
| TERPOS E, 2015, HAEMATOLOGICA | 10.3324/haematol.2014.117176 | 254 | 25.4 | 5.55 |
| CAVO M, 2012, BLOOD | 10.1182/blood-2012-02-408898 | 251 | 19.31 | 3.15 |
| RICHARDSON PG, 2009, BRIT J HAEMATOL | 10.1111/j.1365-2141.2008.07573.x | 248 | 15.5 | 3.02 |
| CAVO M, 2011, BLOOD | 10.1182/blood-2011-02-297325 | 241 | 17.21 | 3.24 |
| HAROUSSEAU JL, 2006, HAEMATOLOGICA |  | 237 | 12.47 | 1.37 |
| RICHARDSON PG, 2013, BLOOD-a | 10.1182/blood-2013-01-481325 | 235 | 19.58 | 5.5 |
| MOREAU P, 2011, BLOOD | 10.1182/blood-2011-05-355081 | 234 | 16.71 | 3.14 |
| RICHARDSON PG, 2009, J CLIN ONCOL | 10.1200/JCO.2008.18.3087 | 221 | 13.81 | 2.69 |
| KUMAR SK, 2014, LANCET ONCOL | 10.1016/S1470-2045(14)71125-8 | 215 | 19.55 | 3.49 |
| DOU QP, 2014, CURR CANCER DRUG TAR | 10.2174/1568009614666140804154511 | 214 | 19.45 | 3.48 |
| CAVALETTI G, 2007, EXP NEUROL | 10.1016/j.expneurol.2006.11.010 | 213 | 11.83 | 1.84 |
| MOREAU P, 2016, BLOOD | 10.1182/blood-2016-01-693580 | 208 | 23.11 | 5.2 |
| SONNEVELD P, 2013, J CLIN ONCOL | 10.1200/JCO.2012.48.4626 | 207 | 17.25 | 4.85 |

**Supplementary Table 6 Results for keyword co-occurrence networks**

| KEYWORD | OCCURRENCES | TOTAL LINK STRENGTH |
| --- | --- | --- |
| therapy | 141 | 680 |
| peripheral neuropathy | 148 | 676 |
| bortezomib | 159 | 667 |
| dexamethasone | 100 | 519 |
| thalidomide | 91 | 509 |
| stem-cell transplantation | 97 | 509 |
| multiple-myeloma | 134 | 491 |
| combination | 89 | 487 |
| trial | 85 | 453 |
| lenalidomide | 78 | 428 |
| survival | 79 | 418 |
| chemotherapy | 66 | 336 |
| prednisone | 53 | 334 |
| melphalan | 52 | 313 |
| neurotoxicity | 66 | 300 |
| efficacy | 50 | 296 |
| reversibility | 48 | 275 |
| plus dexamethasone | 44 | 254 |
| phase-2 | 50 | 237 |
| induced peripheral neuropathy | 65 | 229 |
| open-label | 36 | 216 |
| cancer | 46 | 200 |
| cyclophosphamide | 35 | 198 |
| cells | 45 | 194 |
| phase-iii | 33 | 190 |
| initial treatment | 27 | 187 |
| induction | 34 | 187 |
| proteasome inhibitor bortezomib | 50 | 186 |
| safety | 30 | 173 |
| management | 38 | 171 |
| combination therapy | 27 | 158 |
| elderly-patients | 27 | 155 |
| transplantation | 28 | 155 |
| multicenter | 28 | 151 |
| paclitaxel | 36 | 148 |
| follow-up | 24 | 146 |
| maintenance | 24 | 143 |
| lenalidomide plus dexamethasone | 24 | 142 |
| doxorubicin | 20 | 128 |
| phase-ii | 28 | 127 |
| thalidomide plus dexamethasone | 20 | 127 |
| impact | 24 | 127 |
| pain | 38 | 126 |
| melphalan-prednisone | 19 | 118 |
| pegylated liposomal doxorubicin | 17 | 115 |
| risk | 24 | 113 |
| irreversible inhibitor | 20 | 106 |
| quality-of-life | 23 | 104 |
| apoptosis | 29 | 103 |
| single-agent carfilzomib | 15 | 102 |
| induction therapy | 16 | 100 |
| proteasome | 29 | 96 |
| dose dexamethasone | 16 | 96 |
| high-dose therapy | 22 | 95 |
| refractory myeloma | 15 | 91 |
| phase-3 | 17 | 91 |
| consolidation therapy | 15 | 90 |
| diagnosed multiple-myeloma | 16 | 90 |
| apex trial | 14 | 89 |
| autologous transplantation | 15 | 87 |
| randomized phase-iii | 12 | 86 |
| vincristine | 18 | 86 |
| expression | 29 | 84 |
| oxaliplatin | 19 | 84 |
| in-vitro | 18 | 83 |
| activation | 21 | 81 |
| complete response | 15 | 78 |
| extended follow-up | 12 | 78 |
| relapsed multiple-myeloma | 16 | 77 |
| plus melphalan | 12 | 76 |
| refractory multiple-myeloma | 14 | 75 |
| criteria | 14 | 75 |
| induction treatment | 10 | 73 |
| proteasome inhibitor ps-341 | 15 | 71 |
| oral melphalan | 12 | 71 |
| time | 13 | 70 |
| double-blind | 14 | 70 |
| sensitivity | 12 | 69 |
| induced peripheral neurotoxicity | 22 | 69 |
| cisplatin | 13 | 68 |
| carfilzomib | 15 | 68 |
| proteasome inhibitors | 19 | 67 |
| daratumumab | 12 | 67 |
| neuropathy | 21 | 67 |
| questionnaire | 11 | 66 |
| nf-kappa-b | 19 | 66 |
| proteasome inhibitor | 14 | 64 |
| untreated patients | 10 | 63 |
| outcomes | 12 | 62 |
| randomized-trial | 13 | 61 |
| prevention | 13 | 60 |
| pharmacokinetics | 11 | 59 |
| rituximab | 15 | 58 |
| solid tumors | 12 | 58 |
| low-dose dexamethasone | 10 | 58 |
| ps-341 | 15 | 57 |
| antitumor-activity | 10 | 56 |
| mechanisms | 17 | 53 |
| phase-i trial | 12 | 53 |
| of-the-literature | 10 | 51 |
| pr-171 | 10 | 48 |
| inhibition | 14 | 47 |
| guidelines | 10 | 47 |
| risk-factors | 10 | 45 |
| metabolism | 10 | 44 |
| diagnosis | 11 | 41 |
| rat | 15 | 40 |
| non-hodgkins-lymphoma | 10 | 39 |
| disease | 11 | 37 |
| growth | 10 | 37 |
| autophagy | 10 | 35 |
| phase-ii trial | 10 | 32 |
